# Factors Associated with Vaccination Uptake among Young Children: A Follow-Up Study of 1799 Toddlers

**DOI:** 10.3390/vaccines11030535

**Published:** 2023-02-24

**Authors:** Junjie Huang, Calvin K. M. Cheung, Vera M. W. Keung, Amelia S. C. Lo, Sze Chai Chan, Wing Sze Pang, Queenie H. Y. Li, Lancelot W. H. Mui, Albert Lee, Martin C. S. Wong

**Affiliations:** 1 Centre for Health Education and Health Promotion, Faculty of Medicine, The Chinese University of Hong Kong, Hong Kong, China; 2 Jockey Club School of Public Health and Primary Care, Faculty of Medicine, The Chinese University of Hong Kong, Hong Kong, China; 3 School of Public Health, LKS Faculty of Medicine, The University of Hong Kong, Hong Kong, China; 4 The School of Public Health, Peking University, Beijing 100871, China; 5 The School of Public Health, The Chinese Academy of Medical Sciences and The Peking Union Medical Colleges, Beijing 100006, China; 6 The School of Public Health, Fudan University, Shanghai 200433, China

**Keywords:** vaccination, immunization, uptake rate, children, parents, factors

## Abstract

Childhood vaccination is crucial to protect young children from harmful infectious diseases. This study aimed to investigate the recent childhood immunization rate of recommended and additional vaccinations and identify the factors affecting the vaccination uptake of young children in Hong Kong. The self-administrated questionnaires were distributed to parents of toddlers aged 2 to 5. They were asked to provide information on (1) socioeconomic demographic factors; (2) experiences during pregnancy; and (3) the medical history of the toddler. A total of 1799 responses were collected. Children were more likely to be fully vaccinated when they were at a younger age (aOR = 0.61, 95% CI: 0.48–0.78, *p* < 0.001), the first child in the family (aOR second-born = 0.62, 95% CI: 0.48–0.81, *p* < 0.001; aOR third-born = 0.33, 95% CI: 0.19–0.55, *p* < 0.001), had a higher household income (aOR HKD 15,000–HKD 29,999 = 1.80, 95% CI: 1.27–2.55, *p* = 0.001; aOR ≥ HKD 30,000 = 3.42, 95% CI: 2.39–4.90, *p* < 0.001; compared with <HKD 15,000), or with mothers in older age groups (aOR 35–39 years old = 2.45, 95% CI = 1.22–4.93, *p* = 0.012; aOR ≥ 40 = 2.90, 95% CI = 1.24–6.77, *p* = 0.014; compared with ≤ age 24). The uptake of any additional vaccination was 71%. Children who were older (aOR = 1.32, 95% CI: 1.02–1.70, *p* = 0.036), the first child in the family (aOR second-born = 0.74, 95% CI: 0.56–0.99, *p* = 0.043; aOR third-born = 0.55, 95% CI: 0.32–0.96, *p* = 0.034), with higher household income (aOR ≥ HKD 30,000 = 1.61, 95% CI: 1.10–2.37, *p* = 0.016), were exposed to second-hand smoke from the father (aOR: 1.49, 95% CI: 1.08–2.07, *p* = 0.016), experienced hospitalization (twice or more—aOR: 1.44, 95% CI: 1.04–1.99, *p* = 0.027), or were fully vaccinated (aOR: 2.76, 95% CI: 2.12–3.60, *p* < 0.001) were associated with a higher chance of taking an additional vaccine. To encourage the vaccination rate, more attention should be given to families with more children, low-income families, and younger mothers.

## 1. Introduction

Childhood vaccination is crucial to protect young children from harmful infectious diseases. It is estimated that vaccination prevents 3.5–5 million lives lost annually due to vaccine-preventable diseases, including diphtheria, tetanus, pertussis, influenza, and measles [1]. For children, it significantly lowered the infant mortality rate from 6.5% in 1990 to 2.9% in 2018 across the globe [2].

In Hong Kong, the Hong Kong Childhood Immunisation Programme (HKCIP) was introduced in the 1960s [3]. It provides free-of-charge immunization from infants to primary school children, and the vaccination service is provided by the Maternal and Child Health Centres and the outreach school immunization team of the Department of Health [4]. Parents are recommended vaccines for their children at the appropriate time, but they can choose not to vaccinate or partially vaccinate their children according to the schedule, leading to varying vaccination rates. Although vaccines have been proven to be safe and effective in reducing child mortality [2,5], some parents choose not to vaccinate their children. Previous research has found that lower vaccination uptake was significantly related to a higher birth order [6], mothers at a younger age [7], and a lower level of parental trust in vaccination [8]. Additionally, the education level of the parents and socioeconomic level were found to have both positive and negative effects on the childhood vaccination rate in different studies [6,9]. 

However, some studies focused on parental factors, such as their knowledge and attitude, as well as the family features, but did not consider the socioeconomic demographic factors of children [6,8]. To our knowledge, limited research has been conducted on childhood vaccination in the context of Hong Kong [10,11]. Therefore, our study aims to investigate the recent childhood immunization rate of recommended and additional vaccinations and identify the factors affecting the vaccination uptake of young children in Hong Kong.

## 2. Materials and Methods

### 2.1. Subject Recruitment

A total of 1799 children aged 2–5 were recruited from randomly selected local nurseries and kindergartens in each district cluster. Cluster sampling was adopted in which each kindergarten or nursery was considered a cluster. We extracted a full list of kindergartens and nurseries in Hong Kong from the Education Bureau of the Hong Kong SAR Government. The kindergartens and nurseries were then stratified into 18 districts in Hong Kong. Random numbers were generated and schools were selected according to the random numbers. The principals of the selected clusters were contacted for approval of the study participation. All parents with their children studying in the selected schools were invited to participate, and written consent from the parents was obtained. The majority of the subjects were recruited from September 2015 to July 2016, whereas the overall timeframe was from January 2015 to July 2016.

### 2.2. Survey Instruments

The survey was pilot-tested among 149 parents from 4 kindergartens and validated by an expert panel consisting of healthcare professionals, epidemiologists, and physicians. The survey included 17 questions. The caregivers of the toddlers were asked to provide information by self-report on (1) socioeconomic demographic factors: sex of toddlers, age of toddlers and their parents, birth order of the toddlers, monthly household income, and educational background of parents; (2) experiences during pregnancy: maternal alcohol drinking, smoking history of parents, complications/health problems during pregnancy, delivery method, and gestational age at delivery; and (3) medical history of toddlers: previous experience of hospitalization since birth and vaccination record (recommended and additional vaccines). The full questionnaire can be found in Supplementary Material File S1.

### 2.3. Definition of Variables

In this study, the first outcome of full vaccination referred to the uptake of all vaccinations recommended in the Hong Kong Childhood Immunisation Programme (HKCIP) by the Department of Health in Hong Kong. The vaccines recommended include (1) Bacille Calmette–Guerin (BCG) vaccine, (2) hepatitis B vaccine (three doses), (3) diphtheria, tetanus, acellular pertussis, and inactivated poliovirus (DTaP-IPV) vaccine (three doses and booster), (4) pneumococcal vaccine (two doses and booster), (5) varicella vaccine, and (6) measles, mumps, and rubella (MMR) vaccine. The second outcome of additional vaccination referred to the uptake of any of the following vaccines: (1) Rotavirus vaccine, (2) influenza vaccine, (3) varicella vaccine (booster), (4) Haemophilus influenzae type b (Hib) vaccine, (5) combined vaccine, (6) hepatitis A (HepA) vaccine, (7) meningococcal vaccine, or (8) Japanese encephalitis vaccine.

### 2.4. Statistical Analysis

The data were analyzed using IBM Statistical Package for Social Sciences (SPSS) software version 26.0. First, a descriptive analysis of the study participants was conducted. The distribution of participants by various characteristics was illustrated. Furthermore, the uptake rate of various vaccines was presented. For the second part, multiple logistic regression models were set up to examine the association between the explanatory factors and the two outcome variables, namely, (1) fully vaccinated under the recommendation by the Department of Health and (2) having taken any additional vaccines, after adjusting for confounding. The enter method in which all variables were entered at one single time was used. All *p*-values less than 0.05 were considered statistically significant. The ages of the toddlers and their parents appear normally distributed from quantile–quantile (Q-Q) plots and histograms. Although they were found to be statistically significantly deviated from the normal distribution by the Shapiro–Wilk test, it is believed to be caused by the large sample size of the study [12].

## 3. Results

### 3.1. Respondents’ Characteristics

A total of 1799 responses were collected from parents of toddlers of ages 2 to 5 (mean age: 3.42, SD: 0.50; Table 1). Among the toddlers, 53.0% (*n* = 953) and 47.0% (*n* = 846) were boys and girls, respectively. A total of 58.3% (*n* = 962) of toddlers were delivered vaginally and not induced, followed by delivery through Caesarean section (*n* = 552, 33.5%), and vaginally and induced (*n* = 135, 8.2%). More than half of the toddlers (*n* = 972, 59.0%) were delivered during weeks 37–39 of pregnancy, while 35.8% (*n* = 590) and 5.2% (*n* = 85) were delivered during weeks 40 or longer and weeks 36 or less, respectively. A total of 65.3% (*n* = 1079) of the toddlers were the first-born in the family, while around 30% (*n* = 476, 28.8%) were the second-born.

As far as the sociodemographic status of the family is concerned, the majority of the toddlers came from families with a monthly household income of at least HKD 30,000 (*n* = 809, 52.5%). Most of the mothers and fathers were 25–34 years old (mothers: *n* = 1025, 63.1%, mean: 32.79, SD: 4.61; fathers: *n* = 716, 44.9%, mean: 36.11, SD: 6.30), and had completed post-secondary education (mothers: *n* = 804, 49.2%; fathers: *n* = 809, 50.2%).

During pregnancy, 3.0% (*n* = 48) and 2.3% (*n* = 38) of the mothers had a drinking or smoking history, while 24.1% (*n* = 391) of the fathers had smoked. Approximately one-fourth of the mothers (*n* = 411, 24.9%) reported complications/health problems during pregnancy. Since their birth, about 20% of the toddlers (*n* = 377, 21.4%) were hospitalized twice or more.

### 3.2. Prevalence of the Uptake of Vaccination

**Figure 1:**
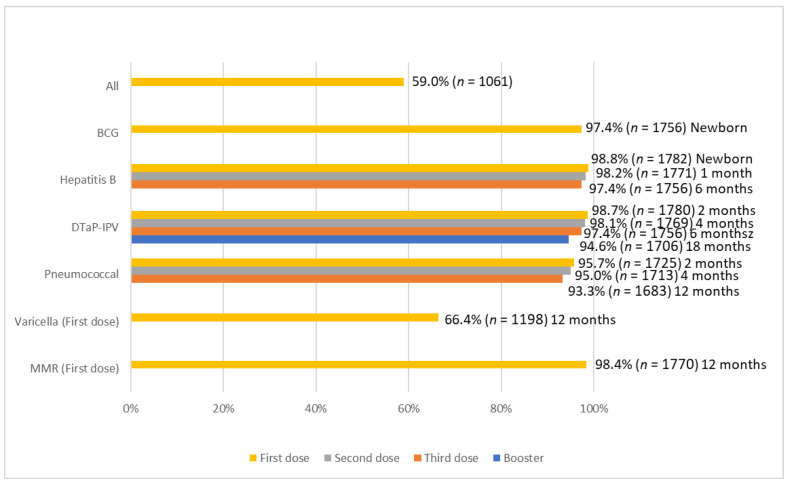
Uptake rate of recommended vaccination.

A total of 59.0% (*n* = 1061) of the toddlers were fully vaccinated based on the recommendation by the Hong Kong Childhood Immunisation Programme (HKCIP). The measles, mumps, and rubella (MMR) vaccine had the highest uptake rate (*n* = 1770, 98.4%), followed by the hepatitis B vaccine (all three doses) (*n* = 1756, 97.4%), the Bacille Calmette–Guerin (BCG) vaccine (*n* = 1756, 97.4%), the diphtheria, tetanus, acellular pertussis, and inactivated poliovirus (DTaP-IPV) vaccine (three doses plus booster) (*n* = 1706, 94.6%), and the pneumococcal vaccine (two doses plus booster) (*n* = 1683, 93.3%). Meanwhile, only 66.4% of the toddlers were vaccinated for varicella (*n* = 1198). Conversely, 1.2% (*n* = 21), 1.3% (*n* = 23), and 4.3% (*n* = 78) had not received any doses of the hepatitis B vaccine, DTap-IPV vaccine, and pneumococcal vaccine, respectively. The vaccination rates of each dose of the vaccine can be found in Figure 1. 

**Figure 2:**
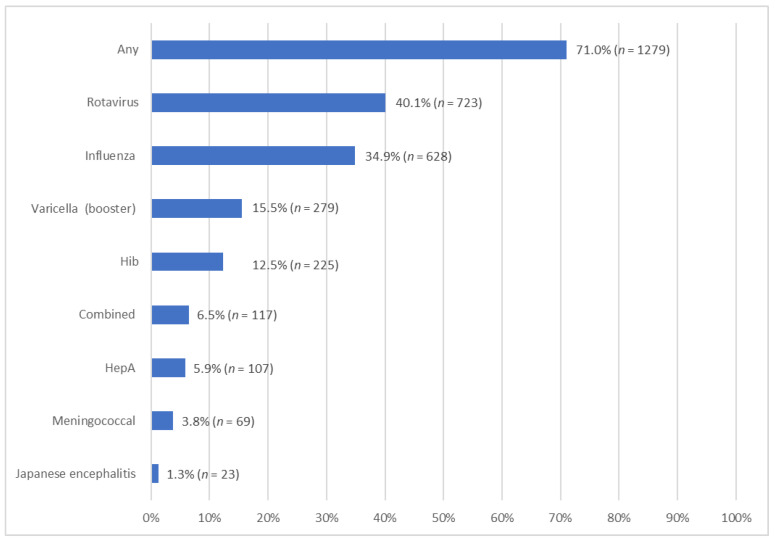
Uptake rate of addition vaccination.

A total of 71.0% (*n* = 1279) of the toddlers had taken at least one additional vaccine. The most prevalent additional vaccine was the rotavirus vaccine (*n* = 723, 40.1%, Figure 2), followed by the influenza vaccine (*n* = 628, 34.9%), the varicella vaccine (booster) (*n* = 279, 15.5%), the Haemophilus influenzae type b (Hib) vaccine (*n* = 225, 12.5%), the combined vaccine (*n* = 117, 6.5%), the hepatitis A (HepA) vaccine (*n* = 107, 5.9%), the meningococcal vaccine (*n* = 69, 3.8%), and the Japanese encephalitis vaccine (*n* = 23, 1.3%).

### 3.3. Factors Associated with Uptake of Vaccination 

Multivariable logistic regression analysis identified several factors significantly associated with the likelihood of full vaccination, as recommended. The age of the toddler was negatively associated with the odds of getting fully vaccinated (aOR = 0.61, 95% CI: 0.48–0.78, *p* < 0.001), i.e., the odds of getting fully vaccinated dropped by 39% per year of age. There was a negative linear relationship between the order of birth and full vaccination. Compared with first-born children, second-born (aOR = 0.62, 95% CI: 0.48–0.81, *p* < 0.001) and third-born (aOR = 0.33, 95% CI: 0.19–0.55, *p* < 0.001) children were significantly less likely to have full vaccination. Meanwhile, weeks of pregnancy (37–39 weeks: aOR = 1.46, 95% CI: 1.14–1.87, *p* = 0.003; compared with 40 weeks or more), monthly household income (HKD 15,000-HKD 29,999: aOR = 1.80, 95% CI: 1.27–2.55, *p* = 0.001; HKD 30,000 or above: aOR = 3.42, 95% CI: 2.39–4.90, *p* < 0.001; compared with below HKD 15,000), and the age of mother (35–39 years old: aOR = 2.45, 95% CI = 1.22–4.93, *p* = 0.012; 40 or above: aOR = 2.90, 95% CI = 1.24–6.77, *p* = 0.014; compared with age 24 or below) were associated with a higher likelihood of full vaccination. The prevalence of full vaccination among toddlers with various characteristics and the aORs can be found in Table 2.

The age of the toddler was positively associated with a higher chance of getting additional vaccinations (aOR = 1.32, 95% CI: 1.02–1.70, *p* = 0.036). Similarly, a negative linear relationship between the birth order and additional vaccination was found: second-born (aOR = 0.74, 95% CI: 0.56–0.99, *p* = 0.043) and third-born (aOR = 0.55, 95% CI: 0.32–0.96, *p* = 0.034) children were significantly less likely to have additional vaccinations compared with first-born children. In contrast, monthly household income (HKD 30,000 or above: aOR = 1.61, 95% CI: 1.10–2.37, *p* = 0.016, compared with below HKD 15,000), father’s smoking during pregnancy (aOR: 1.49, 95% CI: 1.08–2.07, *p* = 0.016), the experience of hospitalization since birth (twice or more—aOR: 1.44, 95% CI: 1.04–1.99, *p* = 0.027), and fully vaccinated based on recommendation (aOR: 2.76, 95% CI: 2.12–3.60, *p* < 0.001) were significantly associated with an increased likelihood of getting additional vaccinations. More detailed results of factors associated with the odds of getting additional vaccinations can be found in Table 3.

## 4. Discussion

### 4.1. Summary of Major Findings

This study assesses various factors that affect the recommended vaccination rate and additional vaccination rate among young children in Hong Kong. The overall uptake rate of all recommended vaccinations was 59%. Over 97% of children had received the Bacille Calmette–Guerin (BCG) vaccine (97.4%) and three doses of the hepatitis B vaccine (97.4%). The average uptake of all other recommended vaccinations was over 90%. However, only 66.4% of children had received the varicella vaccine. Children were more likely to be fully vaccinated when they were at a younger age, the first child in the family, had a higher household income (above HKD 15,000), and had mothers in older age groups.

The uptake of any additional vaccination was 71%. Children who were older, the first child in the family, with higher household income (above HKD 29,999), exposed to second-hand smoke from their father, experienced hospitalization since birth, and were fully vaccinated were associated with a higher chance of receiving an additional vaccine.

### 4.2. Explanations and Comparisons with Previous Literature

The childhood vaccination rate in Hong Kong is relatively high on the global level. According to the World Health Organization (WHO), the global vaccination coverage with three doses of hepatitis B was estimated at 80%; three doses of Pneumococcal vaccination, 51%; and the first dose of measles, 81% [13]. However, it is noted that the coverage rate of the varicella vaccine was significantly lower (66.4%). As suggested by the WHO, at least 80% of varicella vaccination coverage was defined as an adequate level. In Hong Kong, the varicella vaccine was incorporated into the HKCIP in 2014, and children born on or after January 1, 2013, are eligible to receive the varicella vaccine for free [14]. Some of the children included in the current study were born before 2013, and parents need to pay for the varicella vaccine, which might have lowered the incentive to receive the vaccine for this group of people. Additionally, another reason could be parental worry about the varicella vaccine. A previous study found that parents were concerned that taking the varicella vaccine would increase the risk of herpes zoster rather than prevention [15]. As indicated by another study, major reasons for parents not vaccinating their children against varicella were the belief that the infection is not severe enough to warrant vaccination (33.7%), concern about the side effects of vaccination (31.0%), and the thought that the vaccine was ineffective (19.7%) [16].

Concerning additional vaccinations, all vaccines are provided by private clinics only and self-paid, except the booster of the varicella vaccine that has been included in the HKCIP since 2014. The immunization rate of additional vaccinations was generally lower than the global level. For example, the global coverage for the Haemophilus influenzae type b (Hib) vaccine was estimated at 71%, and the rotavirus vaccine was 49% [13], compared with 12.5% for Hib and 40.1% for rotavirus in Hong Kong. As Hong Kong is not a high-risk area for communicable diseases, such as meningococcal infection and Japanese encephalitis [17], the government recommends the general public receive the vaccine before traveling to endemic areas [18,19]. Besides vaccination, there are effective and simple preventive measures against diseases, such as maintaining good personal hygiene and keeping the environment clean [19,20,21].

Our study found that the age of the children and mother, as well as the family household income, had an impact on childhood vaccination uptake. Due to the newly introduced measure of varicella vaccine in the free, comprehensive Childhood Immunization Programme, children born after 2012 can enjoy a free varicella vaccine under HKCIP. No catch-up vaccination is provided by the government at the current stage. Therefore, it is understandable that younger children were more likely to be fully vaccinated. Conversely, our findings illustrated that older children were more likely to receive additional vaccinations. Studies found a tendency of delayed vaccination due to a child’s illness [22] and safety concerns [23]. Parents postpone the vaccination schedule until their children are in a better health condition and grow mentally stronger; this may delay a parental decision to receive additional vaccines. 

Previous studies have presented mixed results on income level and vaccine acceptance. The current study identified that families with higher household incomes (above HKD 15,000) were more likely to get their children fully vaccinated. An American study found that parents with higher household income were more likely to conform with the recommended vaccination schedule (household income: HKD 100,000–HKD 150,000, OR: 0.17, CI: 0.03–0.82, *p* = 0.030, compared with household income <HKD 50,000) [24]. Despite this, several studies found negative [9] and unclear associations [25,26] between family income and vaccine uptake. 

Our findings indicate that younger mothers had a higher vaccine hesitancy. Similar to a prior study, parents at a younger age were associated with a greater level of vaccine hesitancy (b = −0.24, SE = 0.09, *p* = 0.006). However, we only identified the mother’s age as the significant factor but not the father’s age. Mothers are the major caregiver of children in Hong Kong; the vaccination decision is primarily made by the mother. The characteristics of mothers may have a direct effect on vaccinating their children. Another study assessed that mothers aged ≤24 years (OR 0.66, 95% CI: 0.50–0.87) and between 25 and 34 years (OR 0.79, 95% CI: 0.63–0.99) were less likely to fully vaccinate their children [27]. One potential explanation might be that younger mothers use the internet to acquire parenting information [28]; they are more likely to be exposed to misinformation about vaccine safety [29]. They may choose not to receive or only receive some vaccines for their children.

Birth order was well recognized as a key factor affecting childhood vaccination in past studies [6,30,31,32]. A Brazilian study showed that a third- or later-born child was 1.5 times more likely to be not fully vaccinated than the first-born child (aOR: 1.5, 95% CI: 1.3–1.8) [33]. Similar findings were also presented in a Philippines study that having three or more children in a family was significantly associated with incomplete vaccination (OR: 0.6, *p* < 0.05) [31]. Parental investment of time and resources may be decreased with more children [34]. Parents may fail to take later-born children to receive vaccines timely; they may also be less likely to pay for additional vaccines due to resource constraints. In addition, we discovered that children born between 37–39 weeks have an increased chance of being fully vaccinated, but we did not find a significant relationship for premature children (born before 37 weeks). From the previous findings, the immunization coverage was lower among premature children [35], and they are more likely to delay vaccination [36,37,38]. Further studies could be conducted to compare the vaccine uptake of term, preterm, and extremely preterm infants.

Moreover, health-related factors were identified as the major determinants for additional vaccinations, including the experience of hospitalization since birth and the father’s smoking behavior during pregnancy. Exposure to second-hand smoke during pregnancy brings adverse health effects to infants, such as preterm delivery (OR: 1.61, 95% CI: 1.30, 1.99) [39], lower birth weight (OR: −53.7 g, 95% CI: −98.4 to −8.9 g) [40], increased risk of asthma, and poor lung function [41,42]. It is suspected that caregivers recognized the adverse effect of second-hand smoking exposure and attempted to offset the effect by providing additional vaccinations for their children. A study showed that children who had fewer inpatient days (β = 0.22, CI: 0.09–0.53, *p* = 0.02), outpatient visits (β = 0.88, CI: 0.79–0.98, *p* = 0.047), and emergency room visits (β = 0.80, CI: 0.64–0.997, *p* = 0.0006) were more likely to have a refusal of vaccination [9]. The study indicated that healthier children are less likely to receive additional vaccinations, whilst children with poorer health status may receive greater parental health investment, as proved by a previous paper [34]. Without a doubt, fully vaccinated children had a higher chance of receiving additional vaccinations, which implies that parents who had a generally positive attitude towards vaccination were more likely to get additional vaccinations for their children. Previous studies have also supported that parental attitude toward vaccination was important for children to be vaccinated [8]. The vaccine uptake rate was lower if parents were in doubt about the safety and effectiveness of the vaccine [43,44]. Therefore, it is suggested that the government should provide ample information on vaccines to the general public. It is also recommended that vaccination centers be set up in more convenient locations with later service hours to address the difficulty of making time for getting children vaccinated after the long working hours of the parents [45].

### 4.3. Strengths and Limitations

In our study, a thorough summary of the variables associated with childhood immunization of recommended vaccinations and additional vaccinations was provided. An expert panel composed of general practitioners as well as epidemiologists and healthcare specialists validated and pilot-tested the survey. Despite this, there are several limitations to this study. First, the generalization of our study to a larger population should be performed with caution, as the number of responses received from each cluster varied. Second, a cluster effect may exist in our study because subjects from the same nurseries and kindergartens may share some similar characteristics. Additionally, we only examined the correlation between the variables and outcomes, but the underlying cause and effect were not evaluated. We identified that the updated vaccination guideline of HKCIP in 2014 could be one of the possible confounding factors, and other possible confounders need further exploration. Finally, the vaccination record of the toddlers was self-reported by their parents; there might be memory errors or even social-desirability bias. For example, it could be possible that parents falsely report the record, even though toddlers had not received the vaccination, to be viewed favorably. Furthermore, as the current study focuses only on Hong Kong, it may lack external validity, and generalizing results to populations of other countries should be performed with caution.

## 5. Conclusions

The present study concluded that smaller birth order, higher household income, and older age of mothers were associated with full vaccination of their children. Children were more likely to receive additional vaccinations when they are older, the first-born child, have a higher household income, are exposed to second-hand smoke from their father, and were hospitalized. Additionally, children who received all recommended vaccinations were related to a higher chance of taking an additional vaccine. To encourage the vaccination rate, more attention should be given to families with more children, low-income families, and younger age of mothers. The parental attitude and knowledge towards vaccination seem to play an important role in the uptake rate of vaccination, and the government should work on promoting childhood vaccination and dispelling the myth of vaccination. Further studies, including qualitative interviews, can be conducted to explore the barriers and concerns of the parents, while a follow-up study should be conducted to explore the effectiveness of vaccination campaigns. Subsequent vaccination rates should be monitored to capture any changes in parents’ perceptions and decisions on vaccination in the post-COVID-19 era.

## Figures and Tables

**Table 1:** Characteristics of participants in the study.

	*n*	Proportion%
***Sex***		
Female	846	47.0%
Male	953	53.0%
***Age (mean ± SD) *** **3.42 ± 0.50**		
***Delivery***		
Vaginally and not induced (ref)	962	58.3%
Vaginally and induced	135	8.2%
Caesarean section	552	33.5%
***Weeks of pregnancy***		
40 weeks or more (ref)	590	35.8%
37–39 weeks	972	59.0%
36 weeks or less	85	5.2%
***Order of birth***		
First-born (ref)	1079	65.3%
Second-born	476	28.8%
Third-born	97	5.9%
***Monthly household income***		
Below HKD 15,000	260	16.9%
HKD 15,000–HKD 29,999	472	30.6%
HKD 30,000 or above	809	52.5%
***Educational background of mother***		
Primary school or below	33	2.0%
Secondary school	797	48.8%
Post-secondary	804	49.2%
***Educational background of father***		
Primary school or below	39	2.4%
Secondary school	765	47.4%
Post-secondary	809	50.2%
***Age group of mother (mean ± SD) *** **32.79 ± 4.61**		
24 or below	79	4.9%
25–34	1025	63.1%
35–39	422	26.0%
40 or above	98	6.0%
***Age group of father (mean ± SD) *** **36.11 ± 6.30**		
24 or below	24	1.5%
25–34	716	44.9%
35–39	503	31.6%
40 or above	351	22.0%
***Drinking during pregnancy (mother)***		
No	1572	97.0%
Yes	48	3.0%
***Smoking during pregnancy (mother)***		
No	1605	97.7%
Yes	38	2.3%
***Smoking during pregnancy (father)***		
No	1232	75.9%
Yes	391	24.1%
***Complications/health problems during pregnancy***		
No	1240	75.1%
Yes	411	24.9%
***Hospitalization since birth (toddler)***		
Never or 1 time only	1386	78.6%
2 times or more	377	21.4%

**Table 2:** Factors associated with uptake of recommended vaccination.

	*n*	Prevalence %	Adjusted Odd Ratio (aOR)	95% CI	*p*-Value
***Sex***					
Female	505	59.7%	1 (ref)		
Male	556	58.3%	0.939	0.745–1.184	0.595
***Age***			**0.606**	**0.480–0.766**	<0.001 *
***Delivery***					
Vaginally and not induced (ref)	560	58.2%	1 (ref)		
Vaginally and induced	89	65.9%	1.410	0.891–2.230	0.143
Caesarean section	333	60.3%	0.919	0.708–1.194	0.528
***Weeks of Pregnancy***					
40 weeks or more (ref)	314	53.2%	1 (ref)		
37–39 weeks	622	64.0%	**1.459**	**1.136–1.873**	0.003 *
36 weeks or less	47	55.3%	1.181	0.675–2.065	0.561
***Order of birth***					
First-born (ref)	693	64.2%	1 (ref)		
Second-born	251	52.7%	**0.620**	**0.477–0.807**	<0.001 *
Third-born	40	41.2%	**0.326**	**0.192–0.553**	<0.001*
***Monthly household income***					
Below HKD 15,000	101	38.8%	1 (ref)		
HKD 15,000-HKD 29,999	250	53.0%	**1.801**	**1.270–2.553**	0.001 *
HKD 30,000 or above	580	71.7%	**3.423**	**2.389–4.904**	<0.001 *
***Educational background of mother***					
Primary school or below	9	27.3%	1 (ref)		
Secondary school	425	53.3%	2.110	0.759–5.863	0.152
Post-secondary	543	67.5%	2.056	0.724–5.840	0.176
***Educational background of father***					
Primary school or below	15	38.5%	1 (ref)		
Secondary school	415	54.2%	1.624	0.687–3.844	0.270
Post-secondary	538	66.5%	1.674	0.687–4.076	0.257
***Age group of mothers (at toddler’s birth)***					
24 or below	31	39.7%	1 (ref)		
25–34	609	59.5%	1.870	0.993–3.521	0.053
35–39	263	62.3%	**2.449**	**1.217–4.930**	0.012 *
40 or above	60	61.2%	**2.897**	**1.240–6.766**	0.014 *
***Age group of father (at toddler’s birth)***					
24 or below	10	41.7%	1 (ref)		
25–34	437	61.0%	1.587	0.536–4.702	0.404
35–39	304	60.4%	1.264	0.417–3.832	0.679
40 or above	200	57.0%	1.302	0.423–4.010	0.645
***Drinking during pregnancy (mother)***					
No	940	59.8%	1 (ref)		
Yes	30	62.5%	1.019	0.506–2.054	0.958
***Smoking during pregnancy (mother)***					
No	959	59.8%	1 (ref)		
Yes	21	55.3%	2.207	0.914–5.331	0.078
***Smoking during pregnancy (father)***					
No	774	62.8%	1 (ref)		
Yes	198	50.6%	0.960	0.713–1.292	0.786
***Complications/health problems during pregnancy***					
No	721	58.1%	1 (ref)		
Yes	263	64.0%	1.019	0.777–1.336	0.892
***Hospitalization since birth (toddler)***					
Never or 1 time only	819	59.1%	1 (ref)		
2 times or more	229	60.7%	1.165	0.877–1.547	0.293

* *p* values less than 0.05.

**Table 3:** Factors associated with uptake of additional vaccination.

	*n*	Prevalence %	Adjusted Odd Ratio (aOR)	95% CI	*p*-Value
***Sex***					
Female	599	70.6%	1 (ref)		
Male	680	71.4%	1.138	0.884–1.465	0.317
***Age***			**1.315**	**1.018–1.698**	0.036 *
***Delivery***					
Vaginally and not induced (ref)	694	72.0%	1 (ref)		
Vaginally and induced	92	68.1%	0.646	0.409–1.021	0.061
Caesarean section	399	72.3%	0.778	0.584–1.037	0.087
***Weeks of pregnancy***					
40 weeks or more (ref)	399	67.6%	1 (ref)		
37 – 39 weeks	723	74.2%	1.210	0.938–1.607	0.136
36 weeks or less	62	72.9%	1.907	0.970–3.747	0.061
***Order of birth***					
First-born (ref)	808	74.7%	1 (ref)		
Second-born	318	66.8%	**0.742**	**0.556–0.991**	0.043 *
Third-born	60	61.9%	**0.552**	**0.319–0.955**	0.034 *
***Monthly household income***					
Below HKD 15,000	158	60.3%	1 (ref)		
HKD 15,000-HKD 29,999	314	66.4%	1.143	0.794–1.644	0.473
HKD 30,000 or above	644	79.6%	**1.612**	**1.095–2.372**	0.016 *
***Educational background of mother***					
Primary school or below	24	72.7%	1 (ref)		
Secondary school	529	66.2%	0.542	0.204–1.443	0.344
Post-secondary	625	77.8%	0.630	0.229–1.734	0.371
***Educational background of father***					
Primary school or below	29	74.4%	1 (ref)		
Secondary school	507	66.2%	0.474	0.191–1.179	0.108
Post-secondary	627	77.5%	0.738	0.286–1.906	0.530
***Age group of mothers (at toddler’s birth)***					
24 or below	54	68.4%	1 (ref)		
25–34	735	71.7%	0.968	0.494–1.897	0.925
35–39	307	72.7%	0.972	0.461–2.051	0.941
40 or above	71	72.4%	1.015	0.417–2.473	0.973
***Age group of father (at toddler’s birth)***					
24 or below	15	60.0%	1 (ref)		
25–34	529	73.8%	1.722	0.593–4.996	0.317
35–39	377	74.8%	1.938	0.648–5.795	0.236
40 or above	230	65.7%	1.254	0.414–3.795	0.689
***Drinking during pregnancy (mother)***					
No	1134	72.0%	1 (ref)		
Yes	33	68.8%	0.695	0.334–1.445	0.330
***Smoking during pregnancy (mother)***					
No	1154	71.8%	1 (ref)		
Yes	27	71.1%	1.194	0.463–3.079	0.714
***Smoking during pregnancy (father)***					
No	895	72.6%	1 (ref)		
Yes	277	70.5%	**1.493**	**1.076–2.073**	0.016 *
***Complications/health problems during pregnancy***					
No	884	71.1%	1 (ref)		
Yes	302	73.7%	1.079	0.803–1.451	0.614
***Hospitalization since birth (toddler)***					
Never or 1 time only	979	70.5%	1 (ref)		
2 times or more	279	74.0%	**1.440**	**1.041–1.990**	0.027 *
***Fully vaccinated***					
No	421	57.1%	1 (ref)		
Yes	856	80.8%	**2.763**	**2.121–3.601**	<0.001 *

* *p* values less than 0.05.

## Data Availability

The data that supports the findings of this study are available from the corresponding author, upon reasonable request.

## Supplementary Materials

The following supporting information can be downloaded at: https://www.mdpi.com/article/10.3390/vaccines11030535/s1, Supplementary material File S1: Questionnaire.

## References

[1] World Health Organization (2022). Vaccines and Immunization. https://www.who.int/health-topics/vaccines-and-immunization#tab=tab_1.

[2] Nandi A., Shet A. (2020). Why vaccines matter: Understanding the broader health, economic, and child development benefits of routine vaccination. Hum. Vaccines Immunother..

[3] The Government of the Hong Kong Special Administrative Region LCQ13: Childhood Immunisation Programme. Press Releases. https://www.info.gov.hk/gia/general/200802/20/P200802200145.htm.

[4] Family Health Service Child Health. Schedule of Hong Kong Childhood Immunisation Programme. Sep 2022. https://www.fhs.gov.hk/english/main_ser/child_health/child_health_recommend.html.

[5] World Health Organization Vaccines and Immunization: Myths and Misconceptions. Newsroom. Oct 2020. https://www.who.int/news-room/questions-and-answers/item/vaccines-and-immunization-myths-and-misconceptions.

[6] de Cantuária Tauil M., Sato A.P.S., Waldman E.A. (2016). Factors associated with incomplete or delayed vaccination across countries: A systematic review. Vaccine.

[7] Cotter J.J., Bramble J.D., Bovbjerg V.E., Pugh C.B., McClish D.K., Tipton G., Smith W.R. (2002). Timeliness of immunizations of children in a Medicaid primary care case management managed care program. J. Natl. Med. Assoc..

[8] Smith L.E., Amlôt R., Weinman J., Yiend J., Rubin G.J. (2017). A systematic review of factors affecting vaccine uptake in young children. Vaccine.

[9] Wei F., Mullooly J.P., Goodman M., McCarty M.C., Hanson A.M., Crane B., Nordin J.D. (2009). Identification and characteristics of vaccine refusers. BMC Pediatrics.

[10] Lau Y.L., Wong W.H.S., Hattangdi-Haridas S.R., Chow C.B. (2020). Evaluating impact of school outreach vaccination programme in Hong Kong influenza season 2018–2019. Hum. Vaccines Immunother..

[11] Wong W.H.-S., Peare S., Lam H.Y., Chow C.B., Lau Y.L. (2022). The estimated age-group specific influenza vaccine coverage rates in Hong Kong and the impact of the school outreach vaccination program. Hum. Vaccines Immunother..

[12] Rochon J., Gondan M., Kieser M. (2012). To test or not to test: Preliminary assessment of normality when comparing two independent samples. BMC Med. Res. Methodol..

[13] World Health Organization Immunization Coverage. Jul 2022. https://www.who.int/news-room/fact-sheets/detail/immunization-coverage.

[14] The Government of the Hong Kong Special Administrative Region Chickenpox Vaccine to Incorporate into DH Childhood Immunisation Programme on July 2. Press Release. June 2014. https://www.info.gov.hk/gia/general/201406/24/P201406240290.htm.

[15] Hagemann C., Streng A., Kraemer A., Liese J.G. (2017). Heterogeneity in coverage for measles and varicella vaccination in toddlers–analysis of factors influencing parental acceptance. BMC Public Health.

[16] Huber A., Gazder J., Dobay O., Mészner Z., Horváth A. (2020). Attitudes towards varicella vaccination in parents and paediatric healthcare providers in Hungary. Vaccine.

[17] Centre for Health Protection Number of Notifiable Infectious Diseases by Month in 2021. Statistics. Oct 2022. https://www.chp.gov.hk/en/statistics/data/10/26/43/6940.html.

[18] Centre for Health Protection Japanese Encephalitis. Health Topics. Aug 2018. https://www.chp.gov.hk/en/healthtopics/content/24/28.html.

[19] Centre for Health Protection Meningococcal Infection. Health Topics. Sep 2020. https://www.chp.gov.hk/en/healthtopics/content/24/2086.html.

[20] Centre for Health Protection Haemophilus Influenzae Type b Infection. Health Topics. July 2019. https://www.chp.gov.hk/en/healthtopics/content/24/8870.html.

[21] Centre for Health Protection Rotavirus Infection. Health Topics. July 2019. https://www.chp.gov.hk/en/healthtopics/content/24/38.html.

[22] Yawn B.P., Xia Z., Edmonson L., Jacobson R.M., Jacobsen S.J. (2000). Barriers to immunization in a relatively affluent community. J. Am. Board Fam. Pract..

[23] Smith P.J., Humiston S.G., Parnell T., Vannice K.S., Salmon D.A. (2010). The association between intentional delay of vaccine administration and timely childhood vaccination coverage. Public Health Rep..

[24] Brunson E.K. (2013). The impact of social networks on parents’ vaccination decisions. Pediatrics.

[25] De Figueiredo A., Simas C., Karafillakis E., Paterson P., Larson H.J. (2020). Mapping global trends in vaccine confidence and investigating barriers to vaccine uptake: A large-scale retrospective temporal modelling study. Lancet.

[26] Bell C.A., Simmonds K.A., MacDonald S.E. (2015). Exploring the heterogeneity among partially vaccinated children in a population-based cohort. Vaccine.

[27] Antai D. (2012). Gender inequities, relationship power, and childhood immunization uptake in Nigeria: A population-based cross-sectional study. Int. J. Infect. Dis..

[28] Baker S., Sanders M.R., Morawska A. (2017). Who uses online parenting support? A cross-sectional survey exploring Australian parents’ internet use for parenting. J. Child Fam. Stud..

[29] Wang Y., McKee M., Torbica A., Stuckler D. (2019). Systematic literature review on the spread of health-related misinformation on social media. Soc. Sci. Med..

[30] Babirye J.N., Engebretsen I.M., Makumbi F., Fadnes L.T., Wamani H., Tylleskar T., Nuwaha F. (2012). Timeliness of childhood vaccinations in Kampala Uganda: A community-based cross-sectional study. PLoS ONE.

[31] Bondy J.N., Thind A., Koval J.J., Speechley K.N. (2009). Identifying the determinants of childhood immunization in the Philippines. Vaccine.

[32] Bobo J.K., Gale J.L., Thapa P.B., Wassilak S.G. (1993). Risk factors for delayed immunization in a random sample of 1163 children from Oregon and Washington. Pediatrics.

[33] Barata R.B., de Almeida Ribeiro M.C.S., de Moraes J.C., Flannery B., Vaccine Coverage Survey 2007 Group (2012). Socioeconomic inequalities and vaccination coverage: Results of an immunisation coverage survey in 27 Brazilian capitals, 2007–2008. J. Epidemiol. Community Health.

[34] Pruckner G.J., Schneeweis N., Schober T., Zweimüller M. (2021). Birth order, parental health investment, and health in childhood. J. Health Econ..

[35] Fathima P., Gidding H.F., Snelling T.L., McIntyre P.B., Blyth C.C., Sheridan S., Liu B., de Klerk N., Moore H.C. (2019). Timeliness and factors associated with rotavirus vaccine uptake among Australian Aboriginal and non-Aboriginal children: A record linkage cohort study. Vaccine.

[36] Slack M., Thwaites R. (2000). Timing of immunisation of premature infants on the neonatal unit and after discharge to the community. Commun. Dis. Public Health.

[37] McKechnie L., Finlay F. (1999). Uptake and timing of immunisations in preterm and term infants. Prof. Care Mother Child.

[38] Sisson H., Gardiner E., Watson R. (2017). Vaccination timeliness in preterm infants: An integrative review of the literature. J. Clin. Nurs..

[39] Khader Y.S., Al-Akour N., AlZubi I.M., Lataifeh I. (2011). The association between second hand smoke and low birth weight and preterm delivery. Matern. Child Health J..

[40] Crane J., Keough M., Murphy P., Burrage L., Hutchens D. (2011). Effects of environmental tobacco smoke on perinatal outcomes: A retrospective cohort study. BJOG Int. J. Obstet. Gynaecol..

[41] Treyster Z., Gitterman B. (2011). Second hand smoke exposure in children: Environmental factors, physiological effects, and interventions within pediatrics. Rev. Environ. Health.

[42] Ngo C.Q., Phan P.T., Vu G.V., Chu H.T., Nguyen T.T., Nguyen M.H., Phan H.T., Ong B.M.Y., Vu G.T., Pham K.T.H. (2019). Prevalence and sources of second-hand smoking exposure among non-smoking pregnant women in an urban setting of Vietnam. Int. J. Environ. Res. Public Health.

[43] Smith P.J., Marcuse E.K., Seward J.F., Zhao Z., Orenstein W.A. (2015). Children and adolescents unvaccinated against measles: Geographic clustering, parents’ beliefs, and missed opportunities. Public Health Rep..

[44] Kaur B. (2011). Attitudes, Risks and Norms: Understanding Parents’ Measles-Mumps-Rubella (MMR) Immunisation Decision-Making. Ph.D. Thesis.

[45] Huang J., Chan S.C., Ko S., Wang H.H.X., Yuan J., Xu W., Zheng Z.-J., Xue H., Zhang L., Jiang J.Y. (2022). Factors Associated with Vaccination Intention against the COVID-19 Pandemic: A Global Population-Based Study. Vaccines.

