# Supplementary material for: Factors Associated with Vaccination Uptake among Young Children: A Follow-Up Study of 1799 Toddlers
Source: Vaccines (Basel). 2023 Feb 24;11(3):535. doi: 10.3390/vaccines11030535 (PMC10053762; doi:10.3390/vaccines11030535)
Supplement: Supplementary file 1 [file vaccines-11-00535-s001.zip › vaccines-2220405-supplementary.pdf]

Supplementary material S1: Questionnaire

1. Sex of the child:

2. Date of birth of the child:

3. How was your child delivered?
A. Vaginally and not induced
B. Vaginally and induced
C. Caesarean section

4. How many weeks pregnant were you when your child was born?
A. 40 weeks or more
B. 37-39 weeks
C. 36 weeks or less

5. Is your child mother’s first born?
A. Yes
B. No, he/ she is the second born
C. No, he/ she is the third born

6. What is the current monthly family income? (in HKD)
A. Below $15000
B. $15000-$29999
C. $30000 or above

7. What is the educational background of the mother?
A. Primary school or below
B. Secondary school
C. Post-secondary

8. What is the educational background of the father?
A. Primary school or below
B. Secondary school
C. Post-secondary

9. Date of birth of the mother:

10. Date of birth of the father:

11. Had mother of the child ever drunk any alcohol during this pregnancy?
Yes

No

12. Had mother of the child ever smoked during this pregnancy?

Yes

No

13. Had father of the child ever smoked during this pregnancy?

Yes

No

14. Throughout this pregnancy, had mother of the child had any complications / health problems which required close monitoring or medical treatment?

Yes

No

15. Since birth, did your child need hospitalization or observation in hospital due to any illness?

A. Never

B. One time

C. Two times or more

16. Did your child receive the following vaccines included in the universal vaccination scheme of Department of Health?

i) BCG Vaccine

ii) Hepatitis B Vaccine 1st Dose

iii) Hepatitis B Vaccine 2nd Dose

iv) Hepatitis B Vaccine 3rd Dose

v) DTaP-IPV Vaccine 1st Dose

vi) DTaP-IPV Vaccine 2nd Dose

vii) DTaP-IPV Vaccine 3rd Dose

viii) DTaP-IPV Vaccine Booster

ix) MMR Vaccine

x) Pneumococcal Vaccine 1st Dose

xi) Pneumococcal Vaccine 2nd Dose

xii) Pneumococcal Vaccine 3rd Dose

xiii) Pneumococcal Vaccine Booster

xiv) Varicella Vaccine

17. Did your child receive the following vaccines not included in the universal vaccination scheme of Department of Health?

i) Influenza vaccine

ii) Haemophilus Influenzae Type B (Hib) vaccine

iii) Meningococcal vaccine

iv) Hepatitis A vaccine

v) Rotavirus vaccine

vi) Varicella vaccine booster dose

vii) Japanese Encephalitis vaccine

viii) Combined vaccines (a combination of various vaccine components)
